# Propofol Inhibits Androgen Production in Rat Immature Leydig Cells

**DOI:** 10.3389/fphar.2019.00760

**Published:** 2019-07-05

**Authors:** Yiyan Wang, Fei Ge, Xiaoheng Li, Chaobo Ni, Keyang Wu, Wenwen Zheng, Yong Chen, Qingquan Lian, Ren-Shan Ge

**Affiliations:** Department of Anesthesiology, The Second Affiliated Hospital and Yuying Children’s Hospital of Wenzhou Medical University, Wenzhou Medical University, Wenzhou, China

**Keywords:** steroidogenesis, Leydig cells, CYP11A1, HSD3B, CYP17A1, propofol

## Abstract

**Background:** Propofol is a widely used anesthetic. Whether propofol inhibits androgen production by rat Leydig cells and the underlying mechanism remains unclear. The objective of the current study was to examine the effects of propofol exposure to rat primary immature Leydig cells and to define propofol-induced inhibition of steroidogenic enzymes in both rat and human testes *in vitro*.

**Methods:** Immature Leydig cells were purified from 35-day-old male Sprague–Dawley rats and were exposed to propofol for 3 h. The androgen production by Leydig cells under basal, luteinizing hormone, 8bromo-cAMP, and steroid-substrate stimulated conditions and gene expression of Leydig cells after exposure to propofol were measured. Immature Leydig cells were treated with propofol for 3 h and switched to propofol-free medium for additional 3 and 9 h to test whether propofol-induced inhibition is reversible. ^3^H-Steroids were used to evaluate the direct action of propofol on cytochrome P450 cholesterol side chain cleavage (CYP11A1), 3β-hydroxysteroid dehydrogenase (HSD3B), cytochrome P450 17α-hydroxylase/17,20-lyase (CYP17A1), and 17β-hydroxysteroid dehydrogenase 3 (HSD17B3) activities in rat and human testes *in vitro*.

**Results:** Propofol significantly lowered luteinizing hormone and 8bromo-cAMP stimulated androgen production by Leydig cells after 3-h exposure. Further investigation showed that propofol down-regulated the expression of *Cyp11a1* and *Cyp17a1* and their proteins at 5 and 50 µM, although it up-regulated *Lhcgr* expression at 50 µM. Propofol significantly suppressed phosphorylation of ERK1/2 and induced ROS production in immature Leydig cells at 5 and 50 µM. Propofol significantly induced apoptosis of immature Leydig cells at 50 µM. Propofol specifically inhibited rat and human testis HSD3B activities *in vitro*. The half maximal inhibitory concentrations of propofol for rat and human HSD3B enzymes were 1.011 ± 0.065 and 3.498 ± 0.067 µM, respectively. The mode of action of propofol of inhibiting HSD3B was competitive when pregnenolone was added. At 50 µM, propofol did not directly inhibit rat and human testis CYP11A1, CYP17A1, and HSD17B3 activities *in vitro*.

**Conclusion:** Propofol inhibits androgen production *via* both directly inhibiting HSD3B activity and down-regulating *Cyp11a1* and *Cyp17a1* expression in Leydig cells. Suppression of steroidogenic enzymes is presumably associated with the lower production of androgen by Leydig cells after propofol treatment. However, propofol-induced inhibition on androgen production is reversible.

## Introduction

Propofol is a commonly used intravenous short-acting anesthetic for the induction and maintenance of general anesthesia. Its safety and possible side effects to patients are of great concern. Studies have revealed that propofol causes damage to neurons in the developing brain in rodents (Tagawa et al., 2014; Chen et al., 2016) and induces addictive behaviors in rodents and humans (Koopmann et al., 2011; Bonnet and Scherbaum, 2012; Lian et al., 2012; Earley and Finver, 2013; Koroglu and Tezcan, 2016; Wang et al., 2016; Wu et al., 2016). Propofol is often used as an analgesic or sedative in humans during *in vitro* fertilization procedures, and it accumulates in the follicular fluid of oocytes (Christiaens et al., 1999; Ben-Shlomo et al., 2000). In mice, propofol inhibits testosterone production by Leydig cells (Robertson et al., 1985). However, its mechanism of action remains largely unknown.

Testosterone level relies upon the pubertal development of Leydig cells and the balance of androgen biosynthesis and metabolism (Ye et al., 2017). In the rat model, the development of Leydig cells experiences a transient increase of 5α-androstanediol (DIOL) level during puberty with the significant elevation of androgen biosynthetic enzymes, including cytochrome P450 cholesterol side chain cleavage (CYP11A1), 3β-hydroxysteroid dehydrogenase (HSD3B), and cytochrome P450 17α-hydroxylase/17,20-lysase (CYP17A1), and 17β-hydroxysteroid dehydrogenase 3 (HSD17B3) as well as the higher levels of androgen-metabolizing enzymes, including steroid 5α-reductase 1 (SRD5A1) and 3α-hydroxysteroid dehydrogenase (AKR1C14) (**Supplementary Figure S1**) (Ge and Hardy, 1998; Teerds and Huhtaniemi, 2015; Ye et al., 2017). These Leydig cells during puberty are designated as immature Leydig cells (Ge and Hardy, 1998).

Immature Leydig cells are responsive to the stimulation of luteinizing hormone (LH), because these cells express LH receptor (LHCGR) (Shan and Hardy, 1992). LH binds LHCGR, activating G-protein, thus increasing the intracellular cAMP level (Ryu et al., 1988). A cAMP signal cascade stimulates the expression of steroidogenic acute regulatory protein (STAR). STAR transports cholesterol from the cytosol into the mitochondrial inner membrane (Tsuchiya et al., 2003), where the initial catalysis of steroidogenesis by CYP11A1 starts to produce the steroid intermediate, 22R-hydroxycholesterol (22R), which is rapidly converted into pregnenolone (P5). P5 is diffused into the neighboring smooth endoplasmic reticulum, where it is converted into progesterone (P4) by HSD3B and P4 is further converted into androstenedione (D4) *via* CYP17A1. Finally, D4 is catalyzed into testosterone by HSD17B3.

The immature Leydig cell is also a good model for studying the effects of propofol on testosterone metabolism because it abundantly contains SRD5A1 and AKR1C14 (Ge and Hardy, 1998) and propofol is used as an anesthetic in adolescents and children too (Cameron et al., 1992). SRD5A1 in immature Leydig cells irreversibly catalyzes testosterone into more potent androgen, dihydrotestosterone (DHT), and this steroid is further rapidly converted into a weak androgen, DIOL, by AKR1C14, and DIOL is secreted by these immature Leydig cells (**Supplementary Figure S1**) (Ge and Hardy, 1998). Whether propofol affects the function of Leydig cells by altering the expression of steroidogenesis-related proteins and by directly inhibiting these enzyme activities is still unknown. Here, we report these effects of propofol on androgen biosynthesis by rat immature Leydig cells.

## Materials and Methods

### Chemicals and Animals

[7-^3^H(N)]-Pregnenolone (^3^H-P5, specificity = 12.6 Ci/mmol), [1,2,6,7-^3^H(N)]-progesterone (^3^H-P4, specificity = 96.6 Ci/mmol), 1-[1-β,2β-^3^H(N)]-androste-4-ene-3,17-dione (^3^H-D4, specificity = 47.0 Ci/mmol), [1,2,6,7-^3^H(N)]-testosterone (specificity = 80.4 Ci/mmol), 5α-[1,2,4,5,6,7-^3^H]-dihydrotestosterone (^3^H-DHT, specificity = 90.0 Ci/mmol) were purchased from DuPont-New England Nuclear (Boston, MA). Unlabeled 22R, P5, P4, D4, testosterone, DHT, and DIOL were obtained from Steraloids (Newport, RI). Propofol (Cat. No. BP1031) and 8bromo-cAMP (8BR) were purchased from Sigma (St. Louis, MO). Ovine LH was a gift from National Institute of Diabetes and Digestive and Kidney Diseases (NIDDK). Male Sprague–Dawley rats (age of 28 days) were purchased from Shanghai Laboratory Animal Center (Shanghai, China). Human male testes (adult males) were obtained from the National Disease Research Interchange (Philadelphia, PA) and were used only for steroidogenic enzyme sources and were used under the guidance of Clinical Investigation Committee of Wenzhou Medical University Second Affiliated Hospital. All animal procedures were approved by the Institutional Animal Care and Use Committee of Wenzhou Medical University and were performed in accordance with the Guide for the Care and Use of Laboratory Animals.

### Immature Leydig Cell Isolation

Eighteen 28-day-old male rats per isolation were adjusted in the new environment for 7 days. At age of 35 days, they were euthanized by asphyxiation with CO_2_. Rat testes were removed and immature Leydig cells were purified as previously described (Ge and Hardy, 1998). In brief, testis was perfused with M199 medium containing 0.1 mg/ml collagenase *via* the testicular artery. Following digestion with M199 medium containing 0.25 mg/ml collagenase and 0.25 mg/ml DNase for 15 min, cell suspensions were filtered through 100-µm nylon mesh, and the cells were centrifuged under Percoll gradient. The cells with density of 1.070–1.088 g/ml were collected. Purity of immature Leydig cells was evaluated by staining HSD3B activity with 0.4 mM etiocholanolone as the substrate as previously described (Payne et al., 1980). The purity of immature Leydig cells was consistently over 95%. Eight purifications were performed.

### Leydig Cell Culture

To test the effects of propofol on androgen biosynthesis and metabolism, immature Leydig cells were seeded to a 12-well plate at the density of 0.5 × 10^6^ cells/well in DMEM: F12 medium and cultured for 12 h. Then, Leydig cells were switched into DMEM: F12 without (basal) or with hormone (LH, 10 ng/ml) and signalling compound (8BR, 10 mM) and cultured for 3 h in the presence of propofol (5 µM). Propofol was dissolved in dimethyl sulfoxide (DMSO). Enzyme substrates (dissolved in DMSO), including those of CYP11A1 (22R, 20 µM), HSD3B (P5, 20 µM), CYP17A1 (P4, 20 µM), HSD17B3 (D4, 20 µM), SRD5A1 (T, 20 µM), and AKR1C14 (DHT, 20 µM), were also added to immature Leydig cells and cells were cultured for 3 h in the presence of propofol (5 µM). The final concentration of DMSO in the medium was 0.1%, at which DMSO did not affect Leydig cell steroidogenesis. LH acts as a hormone and 8BR acts as a cAMP signalling compound to induce Leydig cell steroidogenesis. Because 8BR can penetrate the cell membrane, therefore it is used to replace the intracellular cAMP. 22R, P5, P4, D4, T, and DHT were used as the respective substrate of the following enzymes: CYP11A1, HSD3B, CYP17A1, HSD17B3, SRD5A1, and AKR1C14. Because 22R can readily penetrate cell membrane and mitochondrial membrane, it is used to replace cholesterol as the substrate for CYP11A1. In another set of experiment, immature Leydig cells were treated with 5 µM propofol for 3 h, then the cells were washed with DMEM: F12 twice and switched to propofol-free DMEM: F12 medium for additional 3 and 9 h. Media were collected for DIOL and testosterone assay after incubation. Cells after treatment with 0, 0.5, 5, and 50 µM propofol under the basal condition were collected for the measurement of Leydig cell mRNA and protein levels.

### Preparation of Mitochondrial, Cytosolic, and Microsomal Proteins

Leydig cell steroidogenic enzymes are present in the mitochondrion (CYP11A1), cytosol (AKR1C14), and microsome (HSD3B, CYP17A1, HSD17B3, and SRD5A1) (Ge and Hardy, 1998). Mitochondrial, cytosolic, and microsomal fractions of rat and human testis samples were used for the steroidogenic enzyme sources to investigate the direct action of propofol on steroidogenic enzyme activities and were prepared as previously described (Ge et al., 1997). Testes (from 35-day-old Sprague–Dawley male rats or adult men) were homogenized in cold 0.01 mM phosphate buffered saline containing 0.25 mM sucrose and centrifuged at 700 × *g* for 30 min. The supernatants were transferred and centrifuged at 10,000 × *g* for 30 min to collect mitochondrial fraction. Supernatants were further centrifuged at 105,000 × *g* for 1 h twice to collect microsomal pellet and the remaining supernatant was used as the cytosolic fraction. Protein concentrations in these fractions were measured using the Bio-Rad Protein Assay Kit (Hercules, CA). Mitochondria were used for CYP11A1 measurement. Microsomes were used for measurement of HSD3B, CYP17A1, HSD17B3, and SRD5A1 activities. Cytosol was used for AKR1C14 assay. The component of the final assay mixture for each enzyme is listed in **Supplementary Table S1**.

### CYP11A1 Assay

CYP11A1 activity in the testicular mitochondria was measured using 22R as the substrate as previously described (Liu et al., 2015). In brief, 22R (2 µM) was dissolved in ethanol, with final ethanol concentration in the reaction solution of less than 0.2%. The substrate concentration was selected based on the Km value of CYP11A1. A 60-min reaction was initiated by adding rat and human testis mitochondria (10 µg protein) in the presence of propofol (50 µM, dissolved in DMSO). The control was 0.2% DMSO and 0.2% ethanol. By the end of incubation, the product, P5, was measured by an RIA kit. The percentage conversion of 22R to P5 was calculated.

### Enzymatic Assays of HSD3B, CYP17A1, HSD17B3, SRD5A1, and AKR1C14

The assays of the testicular microsomal and cytosolic enzymes were performed as previously described (Hu et al., 2009). The detailed conditions for each enzyme assay were listed in the **Supplementary Table S1**. Briefly, the reaction mixture (250 µl) of the substrates (0.2–10 µM), the ^3^H-steroids (60,000 dpm), and co-factors (NAD^+^ or NADPH, 200 µM) were incubated with certain amounts of enzymes (microsomal or cytosolic fractions) for 60–90 min at 34°C (the temperature of the normal testis). For some reactions, propofol was added as an inhibitor (up to 50 µM). By the end of incubations, the reactions were stopped by adding 2 ml ice-cold ether. The steroids were extracted, and the organic layer was dried. Steroids were separated chromatographically on the thin layer plates at the conditions described in **Supplementary Table S1**, and radioactivity was measured by System AR2000 (Bioscan, Washington, DC). The percentage conversion of the substrate into product for each enzyme was calculated.

### Determination of Half Maximum Inhibitory Concentrations (IC_50_) and Mode of Action

The IC_50_ value for HSD3B was determined by adding different concentrations (10 nM−100 µM) of propofol as previously described (Li et al., 2016). The mode of action for HSD3B by propofol was determined by adding various concentrations (10 nM−10 μM) of P5 and 200 μM NAD^+^ in the presence of propofol as previously described (Li et al., 2016). The dose-dependent inhibitions of propofol on rat and human HSD3B activities *in vitro* was subjected to a nonlinear regression by GraphPad version 6 (GraphPad, San Diego, CA), and IC_50_ value was calculated. Lineweaver−Burk plot was used for analyzing the mode of action.

### Assay of P5, DIOL, and Testosterone Concentrations in the Media

P5, DIOL, and testosterone concentrations in the media were measured with a tritium-based radioimmunoassay (RIA) as previously described (Ge and Hardy, 1998) using the commercial RIA kits (IBL, USA). The minimum detection for P5, DIOL, and testosterone was 5 pg. Inter-assay variations of P5, DIOL, and testosterone levels were within 15%.

### Extraction of RNA and Real-Time PCR (RT-qPCR)

Total RNAs were purified from immature Leydig cells after 3-h treatment or after 3- and 9-h recovery experiment using TRIzol reagent (Invitrogen, Carlsbad, CA) as previously described (Lin et al., 2008; Guo et al., 2013). The Leydig cell genes and their primers were listed in **Supplementary Table S2**. The cDNA templates were synthesized by reverse transcription using random hexamers and MMLV reverse transcriptase (Promega, CA). PCR was carried out in a 25-µl reaction mixture with SYBR Green (Bio-Rad Laboratories, Hercules, CA). Reactions were run up to 40 cycles and the melting curves were always checked afterward. The target mRNA levels were adjusted to ribosomal protein S16 (*Rps16*), an internal control. The standard curve method was adopted.

### Western Blot

Immature Leydig cells after 3-h treatment of propofol were homogenized. The protein concentrations were measured using the BCA Protein Assay Kit. Protein samples (30 mg) were loaded and electrophoresed on the 10% polyacrylamide gels, and the proteins were transferred onto the nitrocellulose membrane. The membrane was blocked with 5% bovine serum albumin in TBST buffer for 2 h and incubated with primary antibodies against LHCGR, CYP11A1, CYP17A1, HSD3B1, AKT1, phosphorylated AKT1 (pAKT1), ERK1/2 (ERK), phosphorylated ERK1/2 (pERK), and β-actin (ACTB) at 4°C overnight. Antibody information was listed in **Supplementary Table S3**. The membrane was washed and incubated with HRP-conjugated anti-rabbit or anti-mouse antibodies (1:2,000, Bioword) for 2 h at room temperature. ACTB is a house-keeping protein and it serves as the control. Protein levels were quantified by band intensity using ImageLab software (Bio-Rad Laboratories) and each target protein was normalized to ACTB.

### Measurement of Intracellular Reactive Oxidative Species

Reactive oxidative species (ROS) level was measured with the fluorescence dye 2’7’-dichlorofluorescin diacetate (DCFH-DA) as previously described (Guo et al., 2018). Briefly, 1.5 × 10^5^ cells/ml rat immature Leydig cells were seeded into a six-well plate and cultured in 34°C and 5% CO_2_ incubator. Cells were divided into three groups with or without the treatment of propofol (0, 5, and 50 µM). After 3-h treatment, the adherent cells were harvested and washed with cold PBS, and then cells were resuspended with DCFH-DA (200 µl) for 20 min at 37°C in the dark. Then, fluorescence intensity was measured by flow cytometry.

### Annexin V and PI Assay

Immature Leydig cells were seeded into a 12-well plate with the density of 5 × 10^5^ cells/well and cells were incubated with propofol (0, 5, and 50 µM) for 3 h. To evaluate early and late apoptosis, an Annexin V-FITC/PI Kit was used as previously described (Guo et al., 2018). Cells were harvested and washed with cold PBS and resuspended in 200 µl of Annexin V-binding buffer. Cells were then stained with 5 ml of FITC-labeled Annexin V and 5 ml of PI and instantly measured by flow cytometry.

### Statistics

Data were subjected to analysis by Student’s t-test to identify significant difference whenever two groups (a single concentration of propofol versus control) were compared. Data were subjected to analysis by the Kruskal–Wallis test followed by *ad hoc* Dunnett’s multiple comparisons to identify significant differences between the tested group and the control whenever three or more groups (multiple concentrations of propofol versus control) were compared. All experiments were repeated four to eight times. All data are expressed as means values ± SEM. A difference was regarded as significant at P < 0.05.

#### Results

##### Propofol Inhibits Androgen Production in Immature Leydig Cells

**Figure 1:**
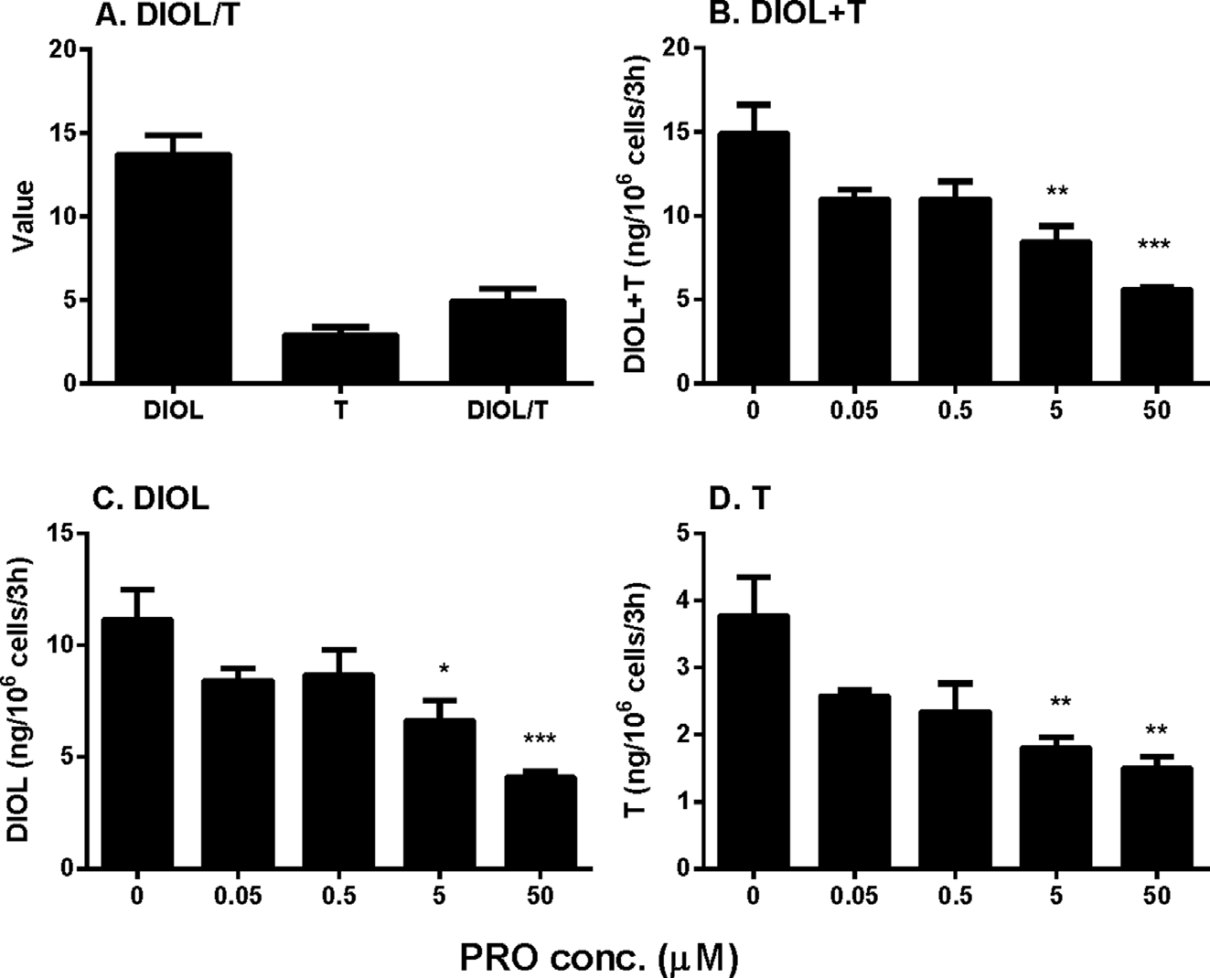
Medium 5α-androstanediol (DIOL) and testosterone (T) levels produced by immature Leydig cells and their ratio (DIOL/T) after 3-h exposure to 0–50 µM propofol (PRO). **(A)** Value stands for ng/10^6^ cells/3 h for DIOL and T levels and value stands for DIOL/T ratio without any treatment; **(B)** DIOL + T levels; **(C)** DIOL levels; **(D)** T levels. Mean values ± SEM, n = 4. Asterisks (*, **, ***) designate significant differences from the control (0 µM PRO) at P < 0.05, 0.01, 0.001, respectively.

Rat immature Leydig cells express CYP11A1, HSD3B, CYP17A1, and HSD17B3 in the androgen biosynthetic cascade as well as SRD5A1 and AKR1C14 in the androgen-metabolizing cascade, thus secreting several androgens, with DIOL being the major one and testosterone the minor one (Ge and Hardy, 1998). Here, we cultured immature Leydig cells in DMEM:F12 medium (basal condition) and found that DIOL level was indeed higher than testosterone level with the DIOL/T (testosterone) ratio about five-folds (**Figure 1A**), confirming the previous finding (Ge and Hardy, 1998). To test the effects of propofol, we treated immature Leydig cells with different concentrations (0.05–50 µM) of propofol for 3 h. Propofol concentration-dependently inhibited total androgen (DIOL+T) levels at ≥ 5 µM (**Figure 1B**). We further analyzed its effects on DIOL (**Figure 1C**) and testosterone (**Figure 1D**) levels, separately, and we found that propofol inhibited both DIOL and testosterone output at ≥5 µM. These results indicate that propofol is able to lower androgen production by immature Leydig cells.

##### Propofol Inhibits Androgen Production in Leydig Cells Under LH and cAMP Stimulations

**Figure 2:**
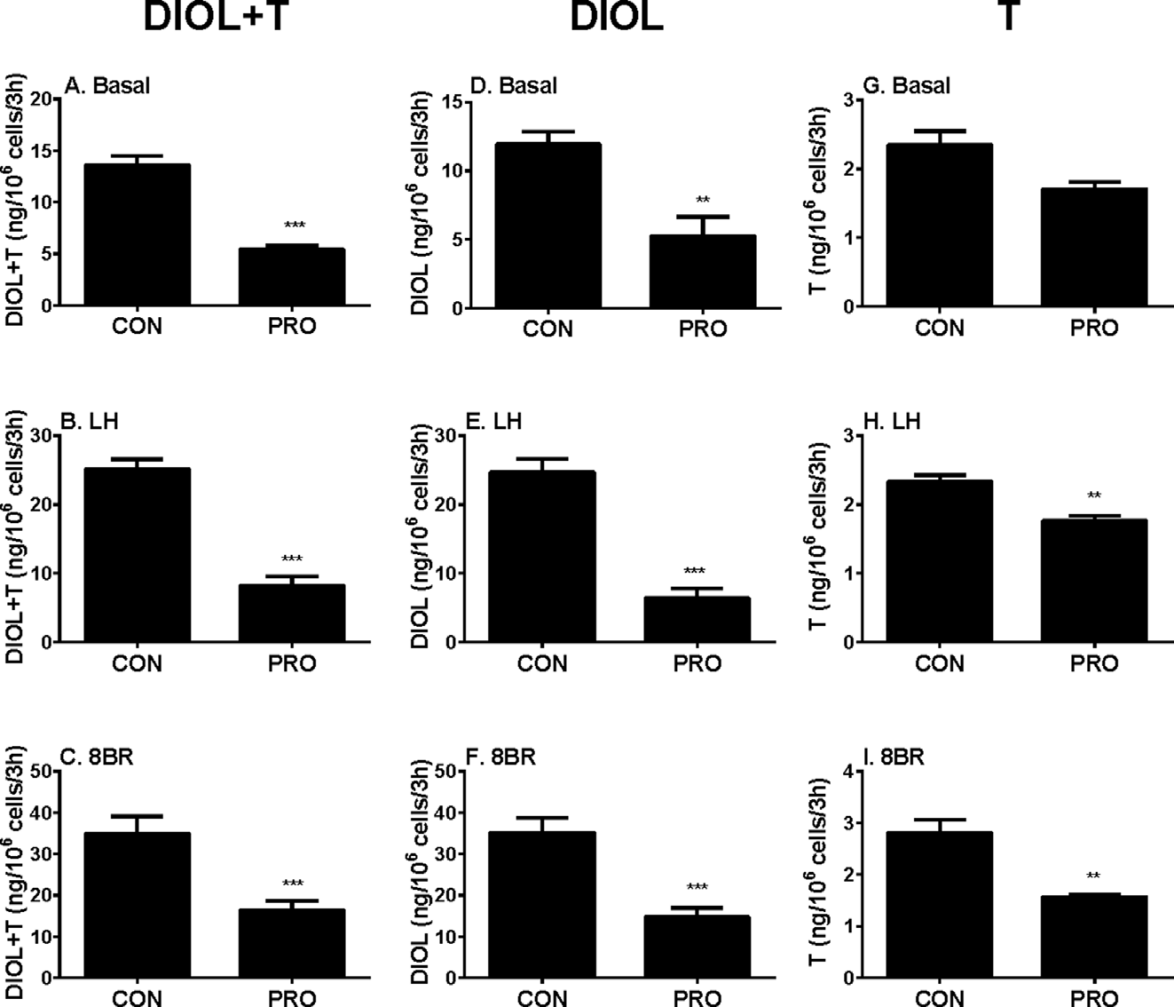
Basal, luteinizing hormone (LH), and 8bromo-cAMP (8BR) stimulated 5α-androstanediol (DIOL) and testosterone (T) levels after 3-h exposure to 5 µM propofol (PRO). **(A–C)** DIOL + T levels; **(D–F)** DIOL levels; **(G–I)** T levels; **(A, D, G)** basal condition; **(B, E, H)** LH stimulation; **(C, F, I)** 8BR stimulation. Mean values ± SEM, n = 4. Asterisks (**, ***) designate significant differences of 5 µM propofol (PRO) from the control (CON, 0 µM propofol) at P < 0.01, 0.001, respectively.

In order to dissect the site(s) of propofol that exerts, we added LH (10 ng/ml) and 8BR (10 mM) to stimulate androgen production by immature Leydig cells and compared them with the control. Indeed, under LH and 8BR stimulations, the medium androgen (DIOL + T) output was much higher than that under the basal condition, indicating that immature Leydig cells respond to LH and 8BR stimulations. We treated Leydig cells under three conditions (basal, LH, and 8BR) together with 5 µM propofol for 3 h. At these three conditions, propofol inhibited DIOL and testosterone outputs (**Figure 2**). The suppressions of androgen levels were comparable between LH and 8BR stimulations, suggesting that the inhibitory site(s) might be beyond the LH signaling cascade.

##### Propofol Inhibits Androgen Biosynthetic Cascade

**Figure 3:**
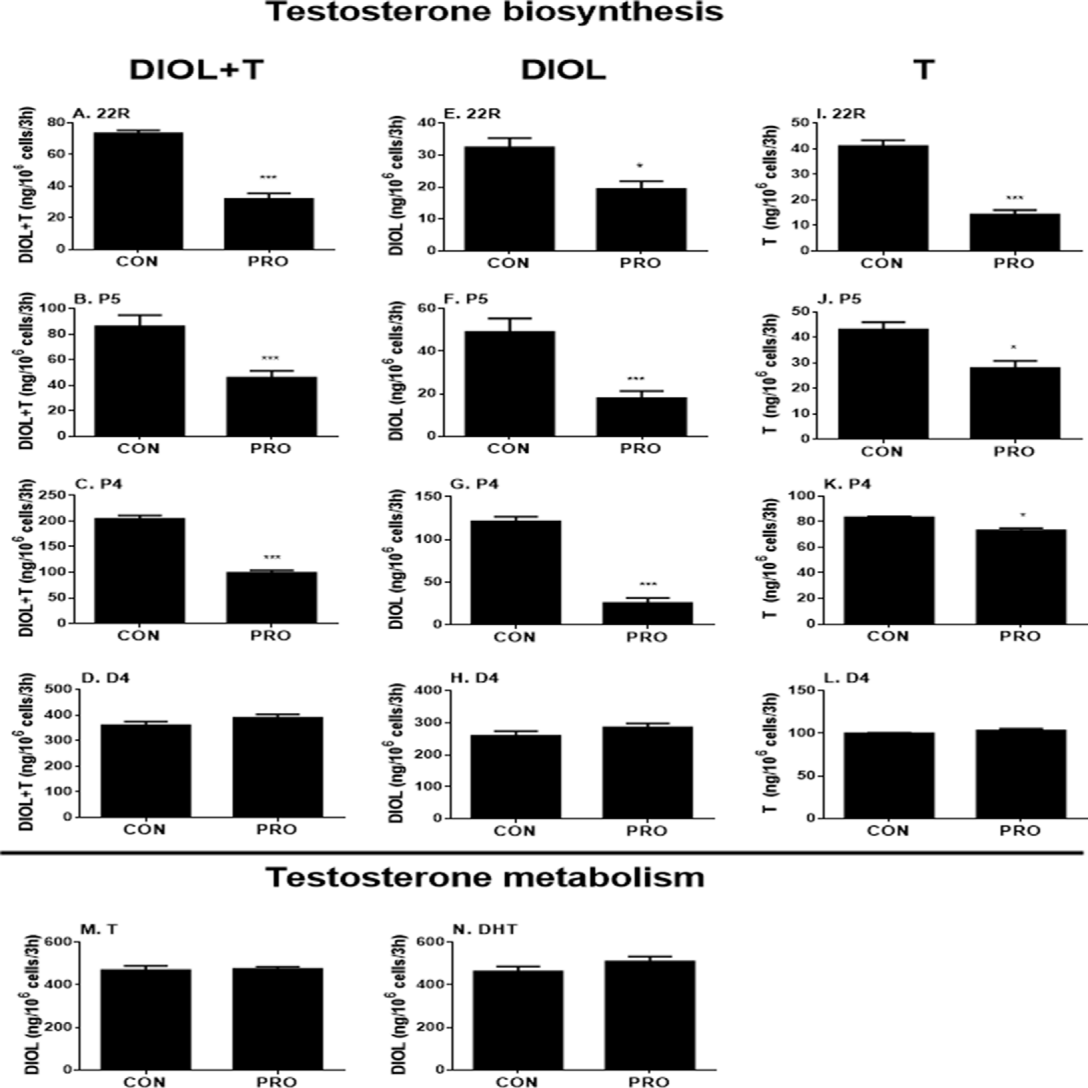
5α-Androstanediol (DIOL) and testosterone (T) levels after 3-h exposure to 5 µM propofol in the presence of various steroid substrates. **(A–D)** DIOL + T levels; **(E–H, M,** and **N**) DIOL levels; **(I–L)** T levels; **(A, E,** and **I)** substrate 22R-hydroxychlosterol (22R); **(B, F,** and **J)** substrate pregnenolone (P5); **(C, G,** and **K)** substrate progesterone (P4); **(D, H,** and **L)** substrate androstenedione (D4); **(M)** substrate testosterone **(T)**; **(N)** substrate dihydrotestosterone **(DHT)**. Mean values ± SEM, n = 4. Asterisks (**, ***) designate significant differences of 5 µM propofol (PRO) from the control (CON) at P < 0.01, 0.001, respectively.

To further explore the specific sites by which propofol might affect androgen outputs in the androgen biosynthetic cascade, we examined all the enzymatic cascades by providing the Leydig cells with different substrates that start the enzymatic reactions from different enzymatic points. After adding 22R, P5, P4, and D4 to Leydig cells as substrates together with 5 µM propofol for 3 h, DIOL and testosterone levels were measured, and the final androgen output (DIOL+T) was still lower with 22R, P5, and P4 (**Figures 3A–C**). However, it was not significantly altered when D4 was used as the substrate (**Figure 3D**). This suggests that the major inhibition by propofol is between the cascades from cholesterol to D4 (CYP11A1, HSD3B1, and CYP17A1). When DIOL and testosterone levels were separately analyzed, propofol showed the similar inhibition (**Figures 3E–L**).

##### Propofol Does Not Inhibit Androgen Metabolizing Cascade

The homeostasis of testosterone in rat immature Leydig cells relies not only on androgen biosynthesis but also on testosterone metabolism. To explore whether propofol affects testosterone metabolism in immature Leydig cells, we examined testosterone-metabolizing cascades by providing Leydig cells with testosterone or DHT. After adding testosterone or DHT to Leydig cells as the substrate together with 5 µM propofol, DIOL levels were not altered after propofol treatment (**Figures 3M, N**), indicating that propofol has no effects on testosterone metabolism.

##### Propofol-Induced Inhibition on Androgen Production Is Reversible

To explore whether propofol-induced inhibition on testosterone production by immature Leydig cells is reversible, we incubated cells with propofol (0 and 5 µM) for 3 h and switched cells to propofol-free medium for additional 3 and 9 h. We observed that 5 µM propofol inhibited androgen production after 3-h treatment and this inhibition was reversible (**Supplementary Figure S2**). This indicates that propofol is a reversible inhibitor of androgen production by immature Leydig cells.

##### Propofol Regulates Some Steroidogenesis-Related Gene Expression

**Figure 4:**
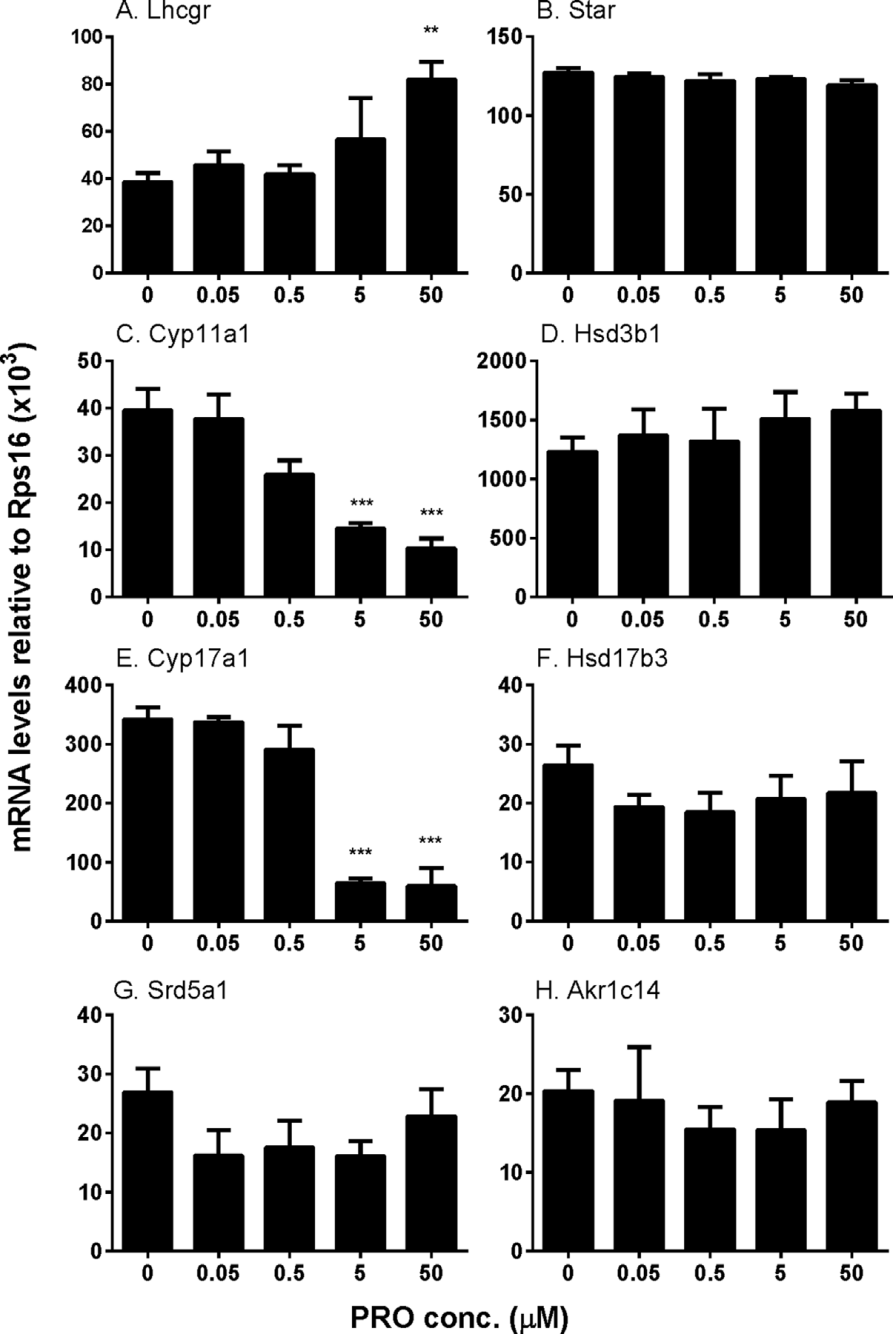
The expression of Leydig cell steroidogenesis-related genes in immature Leydig cells after 3-h exposure to 0–50 µM propofol (PRO). **(A–H)** *Lhcgr*, *Star*, *Cyp11a1*, *Hsd3b1*, *Cyp17a1*, *Hsd17b3*, *Srd5a1*, and *Akr1c14*, respectively. Mean values ± SEM, n = 4. Asterisks (**, ***) designate significant differences from the control (0 µM propofol) at P < 0.01, 0.001, respectively.

We examined the effects of propofol on the expression of steroidogenesis-related genes (**Figure 4**). We found that propofol significantly down-regulated *Cyp11a1* and *Cyp17a1* expression at 5 and 50 µM while it up-regulated *Lhcgr* expression at 50 µM. Propofol did not affect the expression of the other genes. These results indicate that propofol possibly suppresses androgen biosynthesis *via* down-regulating *Cyp11a1* and *Cyp17a1* expression. We further treated cells with propofol (0 and 5 µM) for 3 h and then washed away propofol and incubated cells in propofol-free medium for additional 3 and 9 h and found that the levels of *Cyp11a1* and *Cyp17a1* were comparable to the control (0 µM propofol, **Supplementary Figure S2**). This indicates that propofol reversibly down-regulates *Cyp11a1* and *Cyp17a1*.

##### Propofol Regulates Some Steroidogenesis-Related Protein Expression

**Figure 5:**
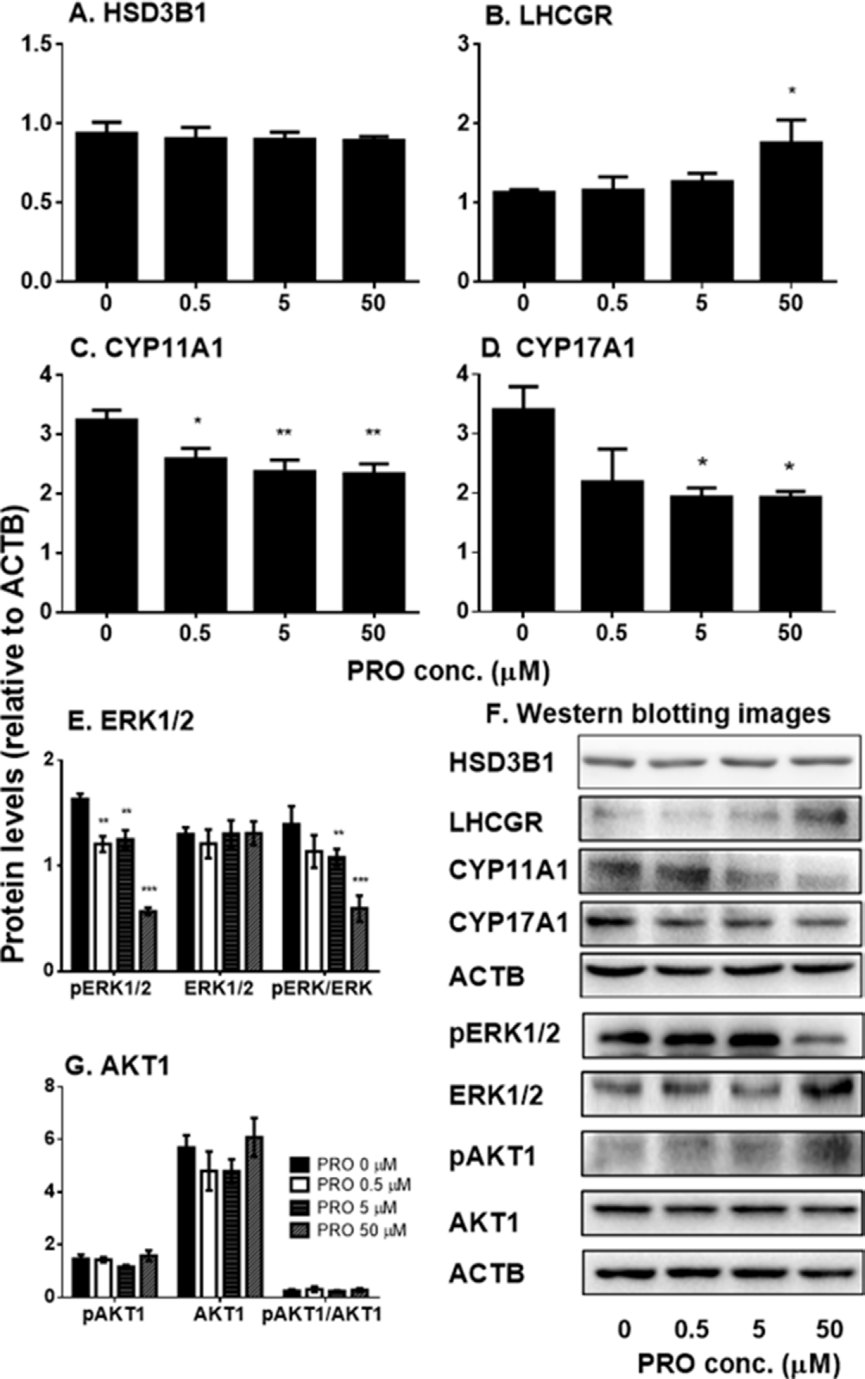
The expression of Leydig cell steroidogenesis-related proteins and ERK1/2 as well as AKT1 phosphorylation in immature Leydig cells after 3-h exposure to 0–50 µM propofol (PRO). **(A–C)** Quantitation of LHCGR, CYP11A1, CYP17A1, and HSD3B proteins; **(D** and **F)** images of Western blot for LHCGR, CYP11A1, CYP17A1, and HSD3B as well as ERK1/2, pERK1/2, AKT1, pAKT1, and ACTB; **(E** and **G)** quantitation of ERK1/2, pERK1/2, pERK1/2 to ERK1/2 (pERK/ERK), AKT1, pAKT1, and pAKT1/AKT1. Mean values ± SEM, n = 4–8. Asterisks (*, **, ***) designate significant differences from the control (0 µM PRO) at P < 0.05, 0.01, and 0.001, respectively.

We also examined the effects of propofol on the levels of steroidogenesis-related proteins (**Figure 5**). We found that propofol significantly lowered Cyp11a1 level at 0.5, 5, and 50 µM and Cyp17a1 level at 5 and 50 µM. However, it increased LHCGR level at 50 µM. Propofol did not affect the level of HSD3B1 protein. This result indicates that propofol inhibits androgen biosynthesis *via* lowering levels of CYP11A1 and CYP17A1 proteins. This confirms that propofol inhibits 22R- and P5-mediated androgen production by immature Leydig cells as shown in **Figure 3**.

##### Propofol Regulates Phosphorylation of AKT1 and ERK1/2

Previous studies have demonstrated that ERK1/2 and AKT1 signaling pathways are involved in Leydig cell steroidogenesis, especially in the expression of *Cyp11a1* and *Cyp17a1* (Gomez et al., 2013; Lai et al., 2014). Therefore, we examined the effects of propofol on phosphorylation of ERK1/2 and AKT1 (**Figure 5**). We found that propofol significantly lowered phosphorylated ERK1/2 (pERK1/2) and the ratio of pERK1/2 to ERK1/2 without affecting ERK1/2 levels at 0.5, 5, and 50 µM. However, propofol did not affect AKT1 and its phosphorylation even at the highest concentration (50 µM). This result indicates that propofol might regulate Leydig cell steroidogenesis mainly *via* interfering with ERK1/2 phosphorylation.

##### Propofol Directly Inhibits Rat and Human HSD3B

**Figure 6:**
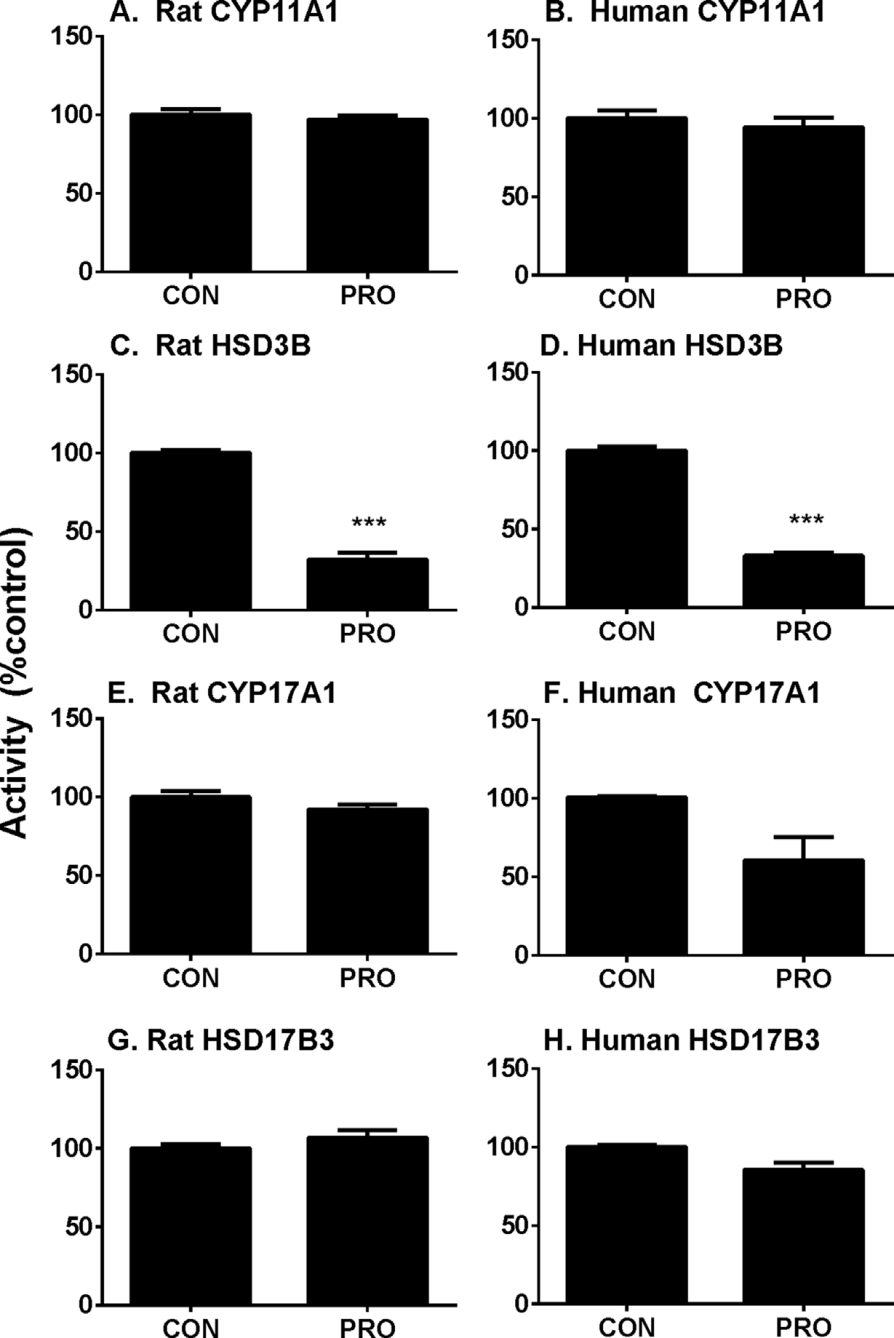
Direct effects of propofol (50 µM) on rat and human testis steroidogenic enzyme activities. Steroidogenic enzyme activities were measured in rat or human testis preparations as described in Materials and Methods and percentage of activities related to the control (CON) was calculated. **(**Panel **A, C, E,** and **G)** rat enzymes; **(**panel **B, D, F,** and **H)** human enzymes; **(**panel **A** and **B)** CYP11A1; **(**panel **C** and **D)** HSD3B; **(**panel **E** and **F)** CYP17A1; **(**panel **G** and **H)** HSD17B3. Mean ± SEM, n = 4. Asterisks (***) designates significant difference from the control (0 µM PRO) at P < 0.001.

**Figure 7:**
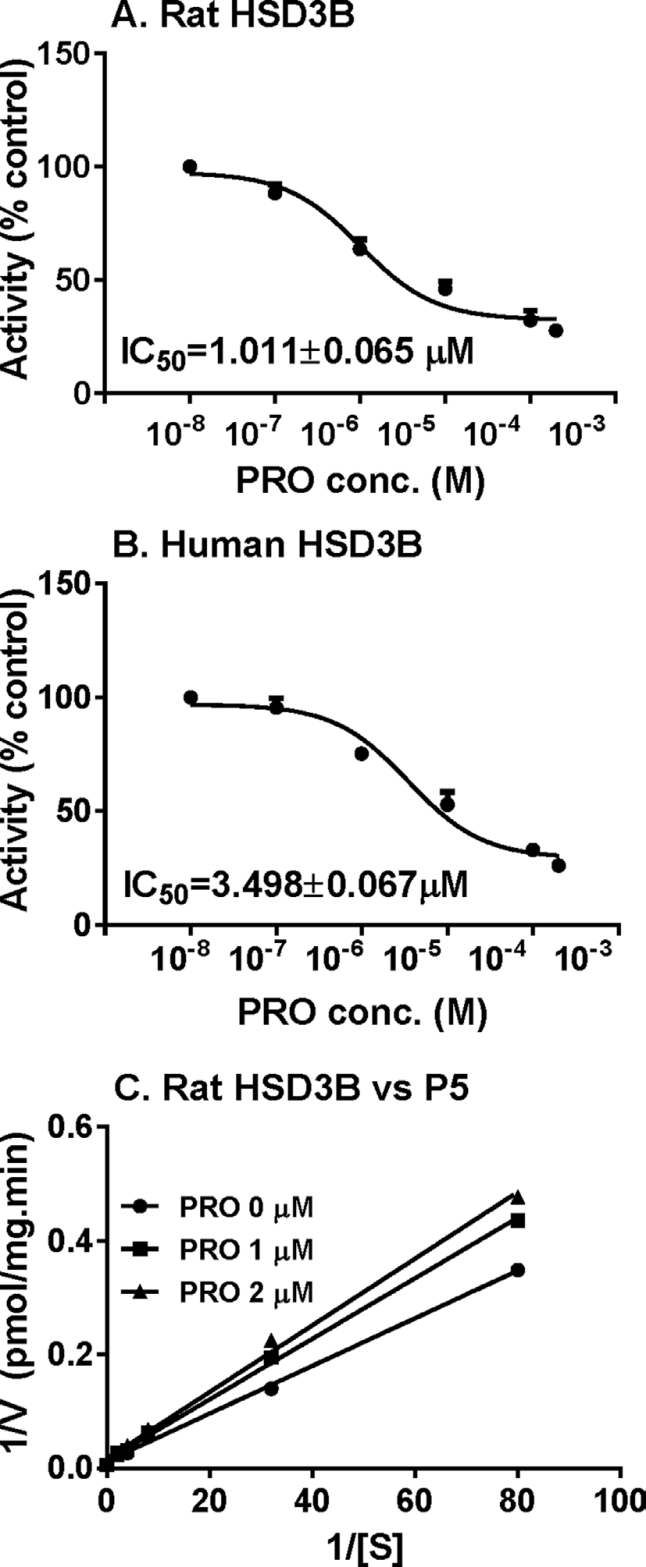
IC_50_ values and mode of action of propofol on rat 3β-hydroxysteroid dehydrogenase (HSD3B). **(**Panel **A** and **B)** IC_50_ values of propofol on rat and human HSD3B, respectively; **(**panel **C)** Lineweaver–Burk plot of rat HSD3B versus pregnenolone (P5) in the presence of propofol. Mean ± SEM, n = 4.

P5-mediated androgen output was decreased after propofol treatment (**Figure 3**). However, the expression of *Hsd3b1*, which encodes HSD3B for this catalysis, and HSD3B1 protein, was not altered by propofol. This prompted us to investigate the direct inhibition of propofol on HSD3B. We used rat and human testes for the sources of HSD3B enzymes. As shown in **Figure 6**, propofol (50 µM) directly inhibited HSD3B activities in both rat and human testicular microsomes. However, propofol (50 µM) did not directly affect other androgen biosynthetic enzyme activities (**Figure 6**). We further investigated the concentration-dependent inhibition of propofol on rat and human testicular HSD3B (**Figure 7**) and found that IC_50_ values of propofol were 1.101 ± 0.065 and 3.498 ± 0.067 µM, respectively (**Figures 7A, B**), suggesting that propofol is a potent testicular HSD3B inhibitor.

##### Propofol Is a Competitive Inhibitor of HSD3B

We further determined that mode of action of propofol on HSD3B. As shown in **Figure 7C**, when P5 was supplied as the substrate, propofol is a competitive inhibitor of rat HSD3B. Propofol also exerted competitive inhibition on human testicular HSD3B (data not shown). This indicates that propofol binds the steroid-binding active site of HSD3B.

##### Propofol Increases ROS Production in Immature Leydig Cells

**Figure 8:**
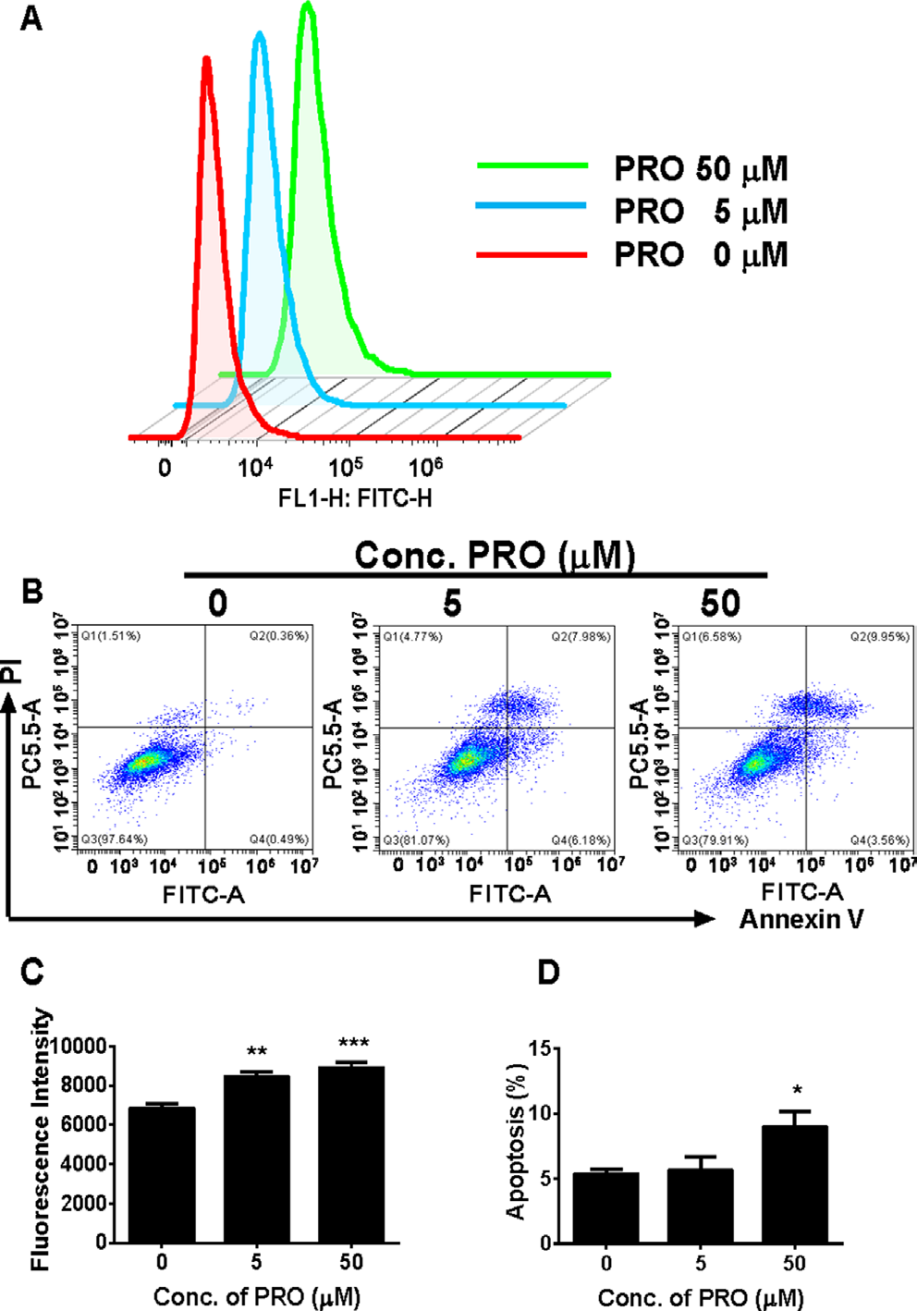
FACS analysis of ROS and annexin V-FITC labeling of apoptosis in Leydig cells after propofol treatment. **(**Panel **A)** count of ROS; **(**panel **B)** FACS spectra for apoptosis after the treatment of 0, 5, and 50 µM PRO; cells in the Q1-LR quadrant were designated apoptotic (PI-negative/annexin V-FITC-positive), cells in Q1-LL were designated living (PI-negative/annexin V-FITC-negative), cells in Q1-UL were designated dead (PI-positive/annexin V-FITC-positive), and cells in Q1-UR were designated damaged (PI-positive/annexin V-FITC-negative); **(**panel **C)** quantitative ROS data; **(**panel **D)** quantitative data for apoptosis analysis. Mean ± SEM, n = 4. Asterisks (*, **, ***) designate significant differences from the control (0 µM PRO) at P < 0.05, 0.01, and 0.001, respectively.

ROS has been found to inhibit androgen production by Leydig cells (Beattie et al., 2013). We analyzed ROS levels in immature Leydig cells after 3-h propofol treatment. As shown in **Figures 8A, C**, propofol significantly increased ROS levels in Leydig cells at 5 and 50 µM. This suggests that propofol induces ROS generation in immature Leydig cells.

##### Propofol Induces Apoptosis of Immature Leydig Cells at the Higher Concentration

We analyzed apoptosis rate of immature Leydig cells after 3-h propofol treatment at two concentrations (5 and 50 µM). Annexin V/PI assay was used to explore the effects of propofol on the apoptosis. Propofol significantly induced apoptosis rate of immature Leydig cells at 50 µM (**Figures 8B, D**). This indicates that propofol induces Leydig cell apoptosis at the higher concentration.

## Discussion

Leydig cells are critical cells in the testis for producing androgens. Low androgen level (androgen deficiency or hypogonadism) induced by some drugs has arisen concerns. Here, we report that propofol, a widely used anesthetic, inhibits androgen production by rat Leydig cells. This drug can directly inhibit HSD3B, a critical androgen-biosynthetic enzyme in both rat and human testis samples. Propofol also down-regulates the key steroidogenic enzyme (*Cyp11a1* and *Cyp17a1*) expression in rat Leydig cells possibly *via* blocking the phosphorylation of ERK1/2.

The production rate of testosterone in the Leydig cells relies on the balance of four critical androgen biosynthetic enzymes (CYP11A1, HSD3B, CYP17A1, and HSD17B3) and two testosterone-metabolizing enzymes (SRD5A1 and AKR1C14) in rat immature Leydig cells. Here, we found that the activities of three (CYP11A1, HSD3B1, and CYP17A1) of four androgen biosynthetic enzymes were lower after propofol treatment, *via* either direct inhibition (such as HSD3B) or down-regulation of gene expression (such as *Cyp11a1* and *Cyp17a1*). However, propofol did not affect androgen metabolizing enzyme (SRD5A1 and AKR1C14) catalysis. Thus, propofol mainly blocks the androgen biosynthetic cascade, resulting in low androgen production. Overall, these results suggest that propofol possibly impacts multiple sites in the androgen biosynthetic cascade, which may contribute to the reduction in androgen secretion.

Interestingly, propofol lowered *Cyp11a1* and *Cyp17a1* mRNA levels at 5 and 50 µM (**Figure 4**). Although it is possible that the reduction of *Cyp11a1* and *Cyp17a1* mRNA levels after 50 µM propofol treatment is partially contributed by the apoptosis of Leydig cells, the propofol-induced apoptosis might insignificantly contribute to them (**Figure 8**). Firstly, the mRNA levels were only measured in live cells not dead or apoptotic cells. Secondly, not all Leydig cells mRNA levels were affected by propofol, only *Cyp11a1* and *Cyp17a1* being lower but *Lhcgr* actually being higher after 50 µM propofol treatment (**Figure 4**). Thirdly, *Cyp11a1* and *Cyp17a1* mRNA levels were also significantly decreased at 5 µM at which propofol did not affect Leydig cell apoptosis (**Figure 8**). However, propofol can lower CYP11A1 protein level at as low as 0.5 µM (**Figure 5**). This indicates that propofol may also interfere with the translation of CYP11A1. CYP11A1 is a mitochondrial inner membrane enzyme and a rate-limiting step for androgen biosynthesis (Ye et al., 2017). The lower CYP11A1 protein level after propofol treatment contributed to the reduced formation of P5 after the supplement of cholesterol intermediate, 22R (**Figure 3**). Interestingly, the down-regulation of *Cyp11a1* and *Cyp17a1* was temporary and reversible (**Supplementary Figure S2**). After additional 3- and 9-h culture in propofol-free medium, the *Cyp11a1* and *Cyp17a1* levels were restored (**Supplementary Figure S2**). Indeed, the androgen production by immature Leydig cells was also recovered (**Supplementary Figure S2**), suggesting that the inhibition of propofol on androgen secretion and gene expression of *Cyp11a1* and *Cyp17a1* is reversible. Propofol lowered *Cyp17a1* mRNA (**Figure 4**) and its protein (**Figure 5**) levels at 5 and 50 µM. CYP17A1 is a smooth endoplasmic reticulum enzyme, which catalyzes two steps of reactions: 17α-hydroxylation and C17,C20-lysis. The lower CYP17A1 protein level after propofol treatment contributed to the decreased formation of D4 after the supplement of P4 substrate (**Figure 3**).

Although the exact mechanisms of regulating *Cyp11a1* and *Cyp17a1* are not clear, only cytochrome P450 enzymes of total seven steroidogenesis-related genes were affected by propofol, suggesting a common regulatory mechanism. One of the possible mechanisms is that propofol blocks the phosphorylation of ERK1/2. Indeed, propofol significantly lowered pERK1/2 and the ratio of pERK1/2 to ERK1/2 at 5 and 50 µM (**Figure 5**).

MEK-ERK1/2 pathway is a critical signaling pathway that mediates many signals from the surface receptors. MEK phosphorylates ERK1/2, activating the down-stream cascades. It has been reported that a Leydig cell conditional double knockout of MEK1/2, the upstream kinases of ERK1/2, induces Leydig cell hypoplasia and the decreased androgen production as well as the down-regulation of steroidogenesis-related genes, including *Cyp17a1*, in mice (Matzkin et al., 2013). Indeed, several hormones and factors, such as LH, epidermal growth factor (EGF), and annexin A5, activate EKR1/2 pathway to stimulate testosterone production. It has been demonstrated that LH can crosstalk with EGF receptor to activate ERK1/2 to stimulate androgen production in Leydig cells (Evaul and Hammes, 2008). Annexin A5 activates ERK1/2 phosphorylation, thus up-regulating *Cyp11a1* (He et al., 2016).

Apparently, AKT1 signaling pathway is not involved in propofol-mediated regulation of steroidogenesis since AKT1 and its phosphorylation were not affected by propofol (**Figure 5**). AKT is a key regulator of Leydig cell development. There are three isoforms of AKT in mammals, AKT1–AKT3. AKT1 is the major isoform in many mammalian tissues and regulates organ development; AKT2 is present in insulin-responsive tissues, regulating glucose metabolism; and AKT3 is mainly expressed in the brain to regulate brain function (Hay, 2011). Knockout of AKT1 in mice causes the testis abnormality (Chen et al., 2001), while double knockout of AKT2–AKT3 in mice does not induce any abnormality of the testis (Dummler et al., 2006). Therefore, AKT1 is a major signaling for the regulation of the testis function. It has been reported that AKT1 is mainly regulated by insulin-like growth factor 1 (Tai et al., 2009), and the knockout of insulin-like growth factor 1 in mice induces the suppression of Leydig cell proliferation (Baker et al., 1996; Hu et al., 2010). Therefore, propofol might not regulate Leydig cell function *via* insulin growth factor 1-AKT1 signaling.

Interestingly, propofol increased *Lhcgr* mRNA level at 50 µM. However, the exact mechanism is still unknown.

**Figure 9:**
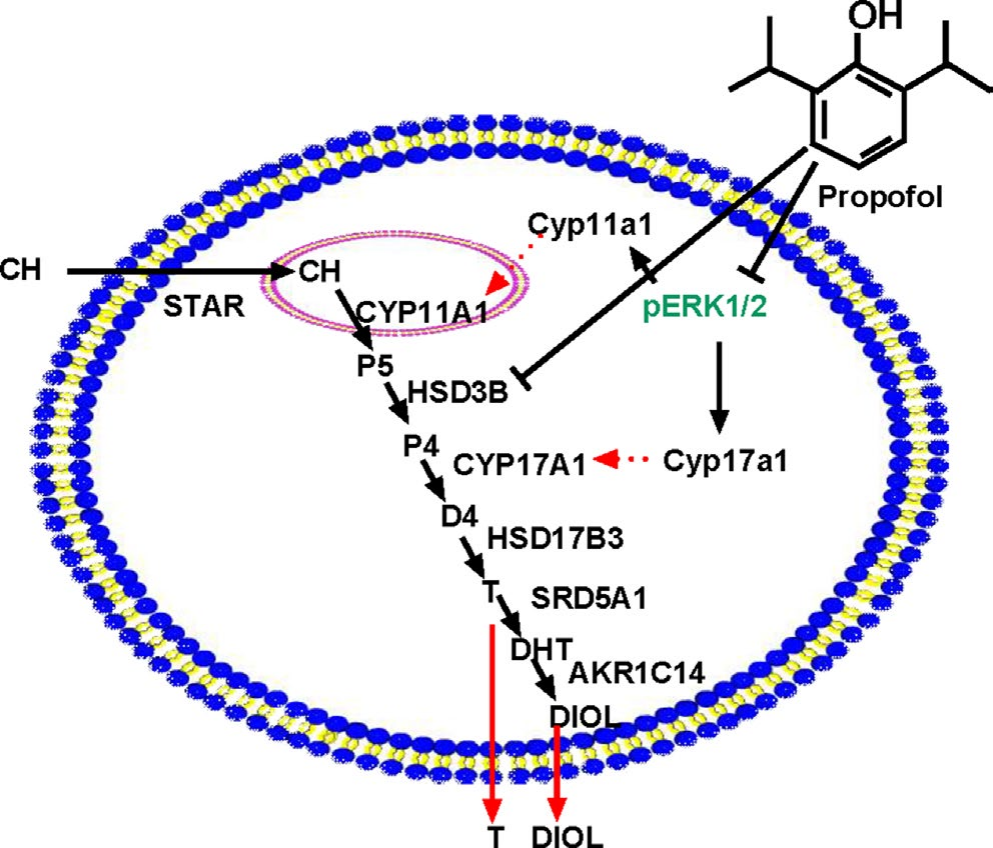
Illustration of the possible mechanisms of propofol in the inhibition of androgen production. PRO blocks ERK1/2 phosphorylation, possibly down-regulating *Cyp11a1* and *Cyp17a1* and thus lowering the enzyme (CYP11A1 and CYP17A1) levels. PRO also directly inhibits HSD3B activity. Therefore, PRO significantly blocks three steps of steroidogenesis in the androgen biosynthetic cascade, resulting in lower androgen (DIOL and T) production. “⊥” = blocking.

HSD3B is a critical enzyme in Leydig cells. HSD3B catalyzes the conversion of the Δ5-3β-hydroxysteroid, P5, into the Δ4-3-ketosteroid, P4, with two sequential chemical reactions: dehydrogenation of P5 in the presence of NAD^+^ and the isomerization of the Δ5-3-keto steroid to yield the Δ4-steroid. Propofol did not affect *Hsd3b1* expression (**Figure 4**) and HSD3B1 protein (**Figure 5**) level in rat immature Leydig cells. Propofol directly inhibited HSD3B activities in both rat and human testis samples (**Figure 7**), suggesting a similar influence of propofol on both rat and human testicular androgen biosynthesis. Propofol competitively inhibited HSD3B for steroid substrate, P5. This suggests that propofol binds the active site of steroid-binding pocket of HSD3B. Propofol is a phenol, structurally similar to steroids (**Figure 9**). Many phenol xenochemicals, such as bisphenol A and gossypol, have been reported to directly inhibit both rat and human testis HSD3B activities (Hu et al., 2009; Ye et al., 2011). This indicates that the phenol group in propofol structure might bind the active site of steroid substrate. Given the necessity of HSD3B for androgen biosynthesis, the direct suppression of this enzyme by propofol might contribute to the decrease of testosterone in Leydig cells.

Propofol is a relatively short-acting drug. Because propofol inhibits HSD3B in a competitive mode by competing with endogenous steroid substrates, the propofol-mediated inhibition would only last a few hours while the drug was present. When propofol is metabolized or eliminated, the decrease in androgen production due to the competitive inhibitory effect of propofol on HSD3B action is reversed. Indeed, after washing away of propofol, no further inhibition on androgen production by immature Leydig cells was found (**Supplementary Figure S2**). In this regard, propofol would have minimal human health implications because any drop in androgen production is a result of propofol inhibition of HSD3B. Interestingly, the down-regulation of *Cyp11a1* and *Cyp17a1* by propofol is reversible and its action would be very temporary.

Propofol also significantly increased ROS production in Leydig cells (**Figure 8**). Several studies have demostrated that overproduction of ROS is capable of causing testicular dysfunction (Tseng et al., 2006), inducing germ cell apoptosis (Zhang et al., 2013), and inhibiting androgen production by Leydig cells (Weissman et al., 2005). Mitochondrion is an important organelle that is involved in the steroid biosynthesis and this organelle is sensitive to oxidative stress (Allen et al., 2004; Hales et al., 2005; Li et al., 2017). Oxidative stress can perturb the mitochondrial function (Diemer et al., 2003). Here, we report that propofol significantly induces ROS production by immature Leydig cells at 5 and 50 µM (**Figure 8**). At the higher cocentration (50 µM), propofol also induces Leydig cell apoptosis (**Figure 8**).

The current study investigated *in vitro* effects of propofol on Leydig cell function and has its limitation due to the lack of *in vivo* study. However, it might be difficult to study *in vivo* effect of propofol on Leydig cell function because propofol is a potent anesthetic and is possible to inhibit the neuron activity in the hypothalamus–pituitary axis, thus affecting testosterone secretion. Indeed, a previous study showed that propofol was able to interfere with pituitary cell function (Ya Deau et al., 2003). Other anesthetics, such as pentobarbitone sodium, also inhibit T rises in boars (Hurden et al., 1979). Propofol is widely used in clinics. To maintain the ideal anesthesia under propofol, serum concentrations of 4.05 µg/ml (∼22.8 µM) is required for major surgery and 2.97 µg/ml (∼16.5 µM) for non-major surgery (Shafer et al., 1988). In such concentration range, propofol apparently is capable of inhibiting androgen production by either directly inhibiting HSD3B activity or down-regulating gene expression of *Cyp11a1* and *Cyp17a1*. Whether such suppression also happens to humans deserves further study. However, since the inhibitions by propofol are reversible, the clinical influence should be limited.

## Ethics Statement

All animal procedures were approved by the Institutional Animal Care and Use Committee of Wenzhou Medical University and were performed in accordance with the Guide for the Care and Use of Laboratory Animals.

## Author Contributions

R-SG and QL conceived and designed the experiments. YW, FG, XL, CN, KW, WZ, and YC performed the experiments. R-SG analyzed the data. R-SG and QL wrote the paper.

## Funding

This research was funded by National Natural Science Foundation of China (81730042 ), Health & Family Planning Commission of Zhejiang Province (11-CX29).

## Conflict of Interest Statement

The authors declare that the research was conducted in the absence of any commercial or financial relationships that could be construed as a potential conflict of interest.

The reviewer GH declared a shared affiliation, with no collaboration, with several of the authors, YW, FG, XL, CN, KW, WZ, YC, SRG, QL to the handling editor at the time of review.
