# Supplementary material for: Propofol Inhibits Androgen Production in Rat Immature Leydig Cells
Source: Front Pharmacol. 2019 Jul 5;10:760. doi: 10.3389/fphar.2019.00760 (PMC6624235; doi:10.3389/fphar.2019.00760)
Supplement: Supplementary file 1 [file Table_1.docx]

Supplementary Table S1: Enzymatic assays of 3β-hydroxysteroid dehydrogenase (HSD3B), 17α-hydroxylase/17,20-lyase (CYP17A1), 17β-hydroxysteroid dehydrogenase 3 (HSD17B3), 5α-reductase 1 (SRD5A1), and 3α-hydroxysteroid dehydrogenase (AKR1C14)

| **Enzyme** | **Substrate (μM)** | **Co-factors (mM)** | **Protein (μg)** | **Time (min)** | **Mobile phase** |
| --- | --- | --- | --- | --- | --- |
| HSD3B | P5 (0.2) | NAD^+^(0.2) | microsome (10) | 90 | chloroform: methanol (97:3, v/v) |
| CYP17A1 | P4 (1) | NADPH (0.2) | microsome (20) | 60 | Chloroform: ether (7:1, v/v) |
| HSD17B3 | D4 (0.2) | NADPH (0.2) | microsome (30) | 60 | chloroform: methanol (97:3, v/v) |
| SRD5A1 | T (2) | NADPH (0.2) | microsome (30) | 60 | chloroform: methanol (97:3, v/v) |
| AKR1C14 | DHT (2) | NADPH (0.2) | cytosol (90) | 60 | Diethyl ether: acetone (98:2.,v/v) |

P5 = pregnenolone; P4 = progesterone; D4 = androstenedione; T = testosterone; DHT = dihydrotestosterone
