# Supplementary material for: Propofol Inhibits Androgen Production in Rat Immature Leydig Cells
Source: Front Pharmacol. 2019 Jul 5;10:760. doi: 10.3389/fphar.2019.00760 (PMC6624235; doi:10.3389/fphar.2019.00760)
Supplement: Supplementary file 2 [file Table_2.docx]

**Supplementary Table S2: Primer information**

| **Primer**  **Symbol** | **Gene name** | **Primer direction** | **Sequences (5’to 3’)** | **PCR**  **(bp)** | **Accession** |
| --- | --- | --- | --- | --- | --- |
| Lhcgr | Luteinizing hormone receptor | Forward | CTGCGCTGTCCTGGCC | 103 | NM_012978 |
|  |  | Reverse | CGACCTCATTAAGTCCCCTGAA |  |  |
| Star | Steroidogenic acute regulatory protein | Forward | CCCAAATGTCAAGGAAATCA | 187 | NM_031558 |
|  |  | Reverse | AGGCATCTCCCCAAAGTG |  |  |
| Cyp11a1 | Cholesterol side chain cleavage enzyme | Forward | AAGTATCCGTGATGTGGG | 127 | NM_017286 |
|  |  | Reverse | TCATACAGTGTCGCCTTTTCT |  |  |
| Hsd3b1 | 3β-Hydroxysteroid dehydrogenase 1 | Forward | CCCTGCTCTACTGGCTTGC | 189 | NM_001007719 |
|  |  | Reverse | TCTGCTTGGCTTCCTCCC |  |  |
| Cyp17a1 | P450 17α-hydroxylase/ 17,20-lyase | Forward | TGGCTTTCCTGGTGCACAATC | 90 | NM_012753 |
|  |  | Reverse | TGAAAGTTGGTGTTCGGCTGAAG |  |  |
| Hsd17b3 | 17β-Hydroxysteroid dehydrogenase 3 | Forward | TGAAAGTTGGTGTTCGGCTGAAG | 202 | NM_054007 |
|  |  | Reverse | TGAAAGTTGGTGTTCGGCTGAAG |  |  |
| Srd5a1 | 5α-Reductase 1 | Forward | TCACCAGAGCGAAGCAGC | 92 | NM_017070 |
|  |  | Reverse | CTAAAGCACAAATGGAATCAGTAT |  |  |
| Akr1c14 | 3α-Hydroxysteroid dehydrogenase | Forward | GCAGCGTGGGGTTGTG | 172 | NM_138547  X17665 |
|  |  | Reverse | TGGATGATTGGGATGGTCA |  |  |
| Rsp16 | Ribosome protein S16 | Forward | AAGTCTTCGGACGCAAGAAA | 146 |  |
|  |  | Reverse | 5’TGCCCAGAAGCAGAACAG |  |  |
