# Supplementary material for: Propofol Inhibits Androgen Production in Rat Immature Leydig Cells
Source: Front Pharmacol. 2019 Jul 5;10:760. doi: 10.3389/fphar.2019.00760 (PMC6624235; doi:10.3389/fphar.2019.00760)
Supplement: Supplementary file 3 [file Table_3.pdf]

Supplementary Table S3. Antibody information

Antibody		Species	Vendor (City, State, catalogue)	Dilution
Symbol	Full name			
ACTB	β-Actin	rabbit	Cell Signaling Technology (Danvers, MA)	1:1000
LHCGR	Luteinizing hormone receptor	rabbit	Multi Sciences (Hangzhou, China)	1:1000
CYP11A1	Cholesterol side chain cleavage	rabbit	Cell Signaling Technology (Danvers, MA)	1:1000
CYP17A1	17α-Hydroxylase/17, 20-lyase	rabbit	Santa Cruz (Santa Cruz, CA)	1:1000
HSD3B1	3β-Hydroxysteroid dehydrogenase 1	rabbit	Abcam (San Francisco, CA)	1:1000
ERK1/2	Extracellular regulated protein kinases 1 and 2	mouse	Abcam (San Francisco, CA)	1:1000
pERK1/2	Phosphorylated EKR1/2	mouse	Abcam (San Francisco, CA)	1:5000
AKT1	RAC-α-serine/threonine-protein kinase	rabbit	Abcam (San Francisco, CA)	1:2000
pAKT1	Phosphorylated (S473) AKT1	rabbit	Abcam (San Francisco, CA)	1:5000
