# Supplementary figures and images for: Propofol Inhibits Androgen Production in Rat Immature Leydig Cells
Source: Front Pharmacol. 2019 Jul 5;10:760. doi: 10.3389/fphar.2019.00760 (PMC6624235; doi:10.3389/fphar.2019.00760)

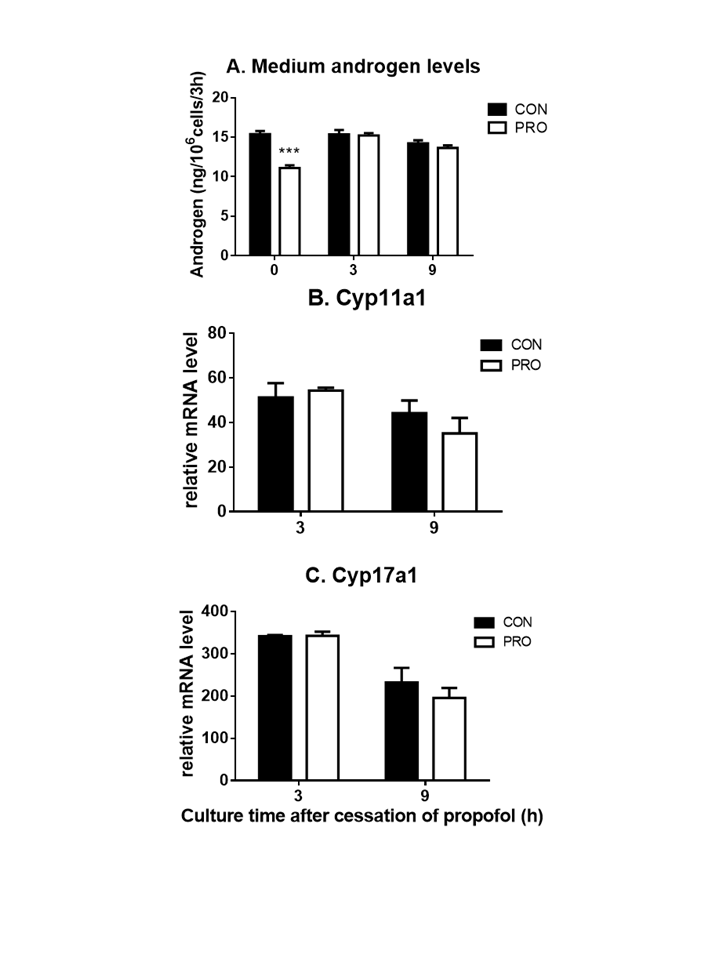

Supplement: Supplementary file 4 [file Image_1.tif]
